# Total Laparoscopic Hysterectomy in Patients with Large Uteri: Comparison of Uterine Removal by Transvaginal and Uterine Morcellation Approaches

**DOI:** 10.1155/2016/8784601

**Published:** 2016-06-22

**Authors:** Haibo Wang, Ping Li, Xiujuan Li, Licai Gao, Caihong Lu, Jinrong Zhao, Ai-ling Zhou

**Affiliations:** Department of Obstetrics and Gynaecology, The 260th Hospital of PLA, Shijiazhuang 050041, China

## Abstract

The aim of this study was to compare the clinical results of total laparoscopic hysterectomy (TLH) for large uterus with uterus size of 12 gestational weeks (g.w.) or greater through transvaginal or uterine morcellation approaches. We retrospectively collected the clinical data of those undergoing total laparoscopic hysterectomies between January 2004 and June 2012. Intraoperative and postoperative outcomes were compared between patients whose large uterus was removed through transvaginal or morcellation approaches. The morcellation group has significantly shorter mean operation time and uterus removal time and smaller incidence of intraoperative complications than the transvaginal group (all *P* < 0.05). No statistical significant difference regarding the mean blood loss, uterine weight, and length of hospital stay was noted in the morcellation and transvaginal groups (all *P* > 0.05). In two groups, there was one patient in each group who underwent conversion to laparotomy due to huge uterus size. With regard to postoperative complications, there was no statistical significant difference regarding the frequencies of pelvic hematoma, vaginal stump infection, and lower limb venous thrombosis in two groups (all *P* > 0.05). TLH through uterine morcellation can reduce the operation time, uterus removal time, and the intraoperative complications and provide comparable postoperative outcomes compared to that through the transvaginal approaches.

## 1. Introduction

Hysterectomy remains the most common major gynecological operation worldwide. It may be carried out by three different routes and its variations: vaginal, abdominal, and laparoscopic [1]. With the advancement of laparoscopic technology, equipment, and training, hysterectomies are increasingly performed laparoscopically [2]. The laparoscopic hysterectomy was firstly performed by Reich et al. [3]. When compared to the open surgery, laparoscopic technique has more advantages with regard to less intraoperative blood loss, decreased length of hospitalization, faster convalescence, fewer complications, less postoperative adhesion formation, and less scar formation [4–6]. The laparoscopic hysterectomy can be categorized into three main types including laparoscopic assisted vaginal hysterectomy (LAVH), laparoscopic supracervical hysterectomy (LSH), and total laparoscopic hysterectomy (TLH). It has become nowadays a preferred choice amongst women who requires hysterectomy for benign gynecological conditions.

According to the previous reports, uterus size above 12 gestational weeks suggests large uterus [7, 8]. A large uterus will lead to several surgical difficulties during laparoscopic hysterectomy, such as limited operative field, restrictive instrument range of motion, and difficult removal of the specimen. The large uteri are often associated with higher risk of complications and morbidities, such as prolonged operation time and excessive blood loss from retrograde bleeding [9–12]. With the progress of surgical techniques and improvements of laparoscopic instruments, hysterectomies for large uteri being performed by laparoscopy are increasingly more safe and effective [1, 6, 13, 14].

Many laparoscopic surgeons have selected the TLH as the surgical procedure, especially because of the recent advances in equipment, surgical techniques, and the advantages for the patients in terms of quick postoperative time to recover. A Cochrane database review in 2006 by Johnson et al. [4] suggests that TLH should be preferred to abdominal hysterectomy for benign gynecological disease. Some authors have demonstrated the feasibility and safety of the TLH for large uterus [1, 6]. During TLH, the uterus sample can be removed through the vagina or through a laparoscopic trocar after morcellation [15]. Despite a number of publications about TLH for large uterus, few surgeons have compared the results of their technique of TLH through the vagina or laparoscopic trocar after morcellation. The aim of this study was to compare the clinical results of TLH for large uterus through transvaginal or uterine morcellation approaches after strict preoperative assessment.

## 2. Patients and Methods

### 2.1. Patients

The study was approved by the institutional review board of 260th Hospital of PLA. Written informed consent for the surgical procedures and the use of personal information for research purposes was obtained from each patient. We retrospectively collected the clinical data of patients undergoing total laparoscopic hysterectomies for larger uteri with uterus size of 12 gestational weeks (g.w.) or greater between January 2004 and June 2012. Inclusion criteria were as follows: (1) patients with uterine myomas or endometrioma; (2) patients who have good physical conditions and have no reproduction requirement; (3) patients with uterine size ≥ 12 weeks of gestation; (4) patients with benign uterine diseases determined by preoperative detection of tumor markers such as alpha-fetoprotein (AFP), carcinoembryonic antigen (CEA), carbohydrate antigen (CA) 125, CA 19-9, and lactate dehydrogenase (LDH); and (5) patients who receive no hormone therapy in the recent 3 months. Exclusion criteria were as follows: (1) patients who are contraindicated to laparoscopic surgery; (2) patients with uterus size >16 g.w.; (3) patients with cervical myoma; (4) patients with uterine myoma associated with ovarian lesions; (5) patients with suspicious malignant gynecological disease diagnosed by ultrasound or MRI; and (6) patients with cervical cancer diagnosed by Thinprip cytologic test (TCT) and malignant endometrial lesions diagnosed by diagnostic curettage.

### 2.2. Preoperative Workup

Vagina was washed once daily using iodophor for 3 consecutive days. Semiliquid diets started 1 day before the operation. Cleansing enema was conducted in the night at 1 day prior to the operation and in the morning at the day of the operation. Indwelling catheter was placed before the operation. All patients underwent physical examination including evaluated detailed clinical history, blood test, electrocardiogram, pelvic and kidney ultrasonography, and thoracic and abdominal X-ray examination. CT or MRI examination was performed to exclude the malignant lesions.

### 2.3. Surgical Technique

#### 2.3.1. Transvaginal Group

Total laparoscopic hysterectomy was performed under endotracheal intubation intravenous anesthesia in a bladder lithotomy position. A four-port laparoscopy was performed after the pneumoperitoneum has been created using a Verres needle. A 10 mm umbilical port was made for laparoscope (Olympus EndoEYE, Olympus Medical Systems, Tokyo, Japan), two 5 mm ports were made for accessory instruments in left and right iliac fossa and one extra 10 mm port was made on left lateral side for 10 mm ligasure. The whole abdominal cavity, including peritoneum, liver, gall bladder, stomach, appendix, and bowels, was inspected for pathologies. After bringing the patient to Trendelenburg position, bowels were moved out of the pelvis and the inner genital organs were inspected. A Cohen uterine manipulator (Karl Storz) with a longer screwed tip was placed through the cervix. Then, uterine manipulator was pushed and tilted slightly to one side. Ultrasound knife was used to cut off round ligament, isthmus portion of the fallopian tube, and the proper ligament of the ovary. The anterior leaf of the broad ligament was dissected. The uterovesical fold is developed, and the bladder is dissected from the uterus. After skeletalisation of the uterine arteries and veins, they were cauterised with bipolar coagulation. The uterine artery divides into ascending and descending parts when it enters the uterus. The uterine vessels were coagulated and dissected on both sides. Ultrasound knife was used to cut off the cardinal ligament of uterus on both sides. Circular colpotomy was performed by using a monopolar knife at the vaginal fornices. After detaching the uterus completely, it was extracted through the vagina by the following methods: segmental resection, split-half resection, and piecemeal resection according to uterus size and shape. After washing the abdominal and pelvic cavity with distilled water, the vaginal stump was sutured using running sutures number 0 Vicryl. Thereafter, running suture was also conducted on the posterior peritoneum. At the end of the operation, thorough inspection of the abdomen was performed to ensure hemostasis. Finally, a drain was inserted via the port-insertion site in the right lower quadrant.

#### 2.3.2. Morcellation Group

The first steps of TLH with morcellation approach were performed as similarly described for transvaginal approach. The distinction occurred in the step of dissecting the uterine artery. After exposing the uterine vessels, the ascending branch of the uterine artery and vein was cauterised with bipolar coagulation. After the uterine body turns purple due to ischemia, a Sawalhe II Supercut*™* morcellator (Karl Storz®, Tuttlingen, Germany) was used through a 10 mm port after augmenting the left lateral assess. Most of the uterine body and myomas were morcellated. Then, routine procedures were used to dissect the uterine vessels, cardinal ligaments, and uterosacral ligaments. Thereafter, a monopolar knife was used to perform a circular colpotomy at the vaginal fornices. The remaining uterus was removed vaginally. The subsequent steps were identical with those in the transvaginal group. In our procedure, uterine body was not separated from uterine neck and thus endoscopic bags were not applied during specimen retrieval.

### 2.4. Postoperative Management

Urethral catheters were routinely removed on the first postoperative day. Semiliquid diets start 24 hours after the operation. Normal diets start according to the conditions of functional recovery of gastrointestinal function. The drain was only removed when it drained less than 50 mL of fluid per 24 h. Patients received anti-infective therapy including penicillin 4 million units iv.bid and metronidazole 0.5 g iv.bid for 3 consecutive days.

### 2.5. Clinical Assessment

All the patients were evaluated by detailed clinical history and physical examination. All patients underwent pelvic and kidney ultrasonography, blood count, and liver and kidney blood tests. The following parameters were evaluated: patient's characteristics (age, weight, body mass index (BMI), parity, and previous surgical history), indications for hysterectomy, operation time, uterus removal time, length of hospital stay, blood loss, uterine weight, and intra- and postoperative complications. Operative time was calculated from the insertion of the trocar to skin closure of the last port site. Estimation of blood loss was made on the volume in the negative pressure suction bottles (mL). Uterus removal time in transvaginal group was calculated from the dissociation of the uterine body to complete removal from the vagina. Uterus removal time in morcellation group was calculated from the dissociation of the uterine body to remove most of the uterine body until the uterus isthmus.

### 2.6. Statistical Analysis

All data were analyzed by using SPSS 11.5 statistical software (SPSS Inc., Chicago, IL). Count data was expressed as number and percentage and compared using *χ* ^2^ or Fisher's exact test. Numeric data are presented as means ± standard deviation (SD) and were compared using *t* tests. A *P* value of less than 0.05 was considered statistically significant.

## 3. Results

### 3.1. Baseline Data

A total of 416 patients with larger uteri treated with total laparoscopic hysterectomies were included in this study. The patients were divided into 2 groups according to the removal approaches of uterine samples: morcellation group (*n* = 254 cases) and transvaginal group (*n* = 162 cases).  Table 1 shows the baseline characteristics in two groups. There was no statistical significance regarding the age, BMI, uterus size, previous cesarean section, uterus dimensions with ultrasound, and indications for TLH between the uterine morcellation and transvaginal groups.

### 3.2. Intraoperative Outcomes

Comparison of intraoperative outcomes between the morcellation and transvaginal groups is shown in Table 2. The mean operation time and uterus removal time were significantly shorter in the morcellation group than in the transvaginal group (116.4 ± 44.6 min versus 128.6 ± 56.4 min and 15.8 ± 6.6 min versus 20.8 ± 7.8 min, all *P* < 0.05). No statistical significant difference regarding the mean blood loss and uterine weight was observed in the morcellation and transvaginal groups (113.2 ± 56.4 mL versus 122.8 ± 61.4 mL and 612.4 ± 143.8 g versus 601.8 ± 138.4 g, all *P* > 0.05). The lengths of hospital stays for the morcellation and transvaginal groups were 3.6 ± 1.6 d and 3.8 ± 1.4 d (*P* > 0.05), respectively. In morcellation group, there was one patient (uterine size: 14 weeks of gestation) who underwent conversion to laparotomy because the patient has adenomyosis with concomitant chronic pelvic inflammation and extensive adhesions in right adnexa uteri and intestinal canal. In transvaginal group, there was one patient (uterine size: 15 weeks of gestation) who underwent conversion to laparotomy due to myoma protruding into the left broad ligament which causes the difficulty of surgical dissection.

With regard to the intraoperative complications, in morcellation group, there were 3 cases of subcutaneous emphysema, 2 cases of intestinal contusions, and 1 case of bladder injury whereas in transvaginal group there were 2 cases of subcutaneous emphysema, 4 cases of intestinal contusions, 4 cases of vaginal stump laceration, 1 case of bladder injury, and 1 case of ureteral injury. The total incidence of intraoperative complications in morcellation group was significantly lower than that in the transvaginal group (2.4% versus 7.5%, *P* < 0.05).

Postoperative histology examination revealed benign uterine leiomyomas or adenomyosis in both groups: 4 women were diagnosed as cellular leiomyoma in morcellation group and 1 woman was diagnosed as cellular leiomyoma in transvaginal group. No undiagnosed uterine malignancies were observed in our series. The 4 cases of cellular leiomyoma in morcellation group have been followed up for 5 years and no abnormality was observed. The 1 case of cellular leiomyoma in transvaginal group has been followed up for 10 years and also no abnormality was observed.

### 3.3. Postoperative Complications

Table 3 shows the postoperative complications. With regard to postoperative complications, there was no statistical significant difference regarding the frequencies of pelvic hematoma (*P* = 0.69), vaginal stump infection (*P* = 1.00), and lower limb venous thrombosis (*P* = 0.82) in two groups. The pelvic hematoma and vaginal stump infection in both groups recovered to normal after being treated with anti-inflammatory therapy and physiotherapy. The patient in the transvaginal group who developed the lower limb venous thrombosis (1 case) recovered to normal after conservative treatment.

## 4. Discussion

TLH is currently accepted as a safe, efficient way to manage benign uterine pathology by doctors and patients and is an acceptable alternative to standard abdominal hysterectomy [16, 17]. With the popularization and advance of the TLH, scholars are increasingly exploring and expanding their surgical scope of application. In the past, large uterus with uterus size above 12 weeks of gestation was considered as the contraindication for laparoscopic hysterectomy due to limited visibility and access to uterine vascular associated with the high risk of complications such as hemorrhage, bowel and urinary injury, difficulty in extracting the uterus, and extended duration of the procedure [18, 19] and was more suitable for laparotomy. With the expansion of the surgical indications of the laparoscopic hysterectomy, there have been a number of publications reporting the TLH for large uterus [20–24]. Vagina is previously considered as the optimal channel of uterine removal. However, for large uterus, it is hard to take out the divided uterus through the narrow vagina and thus measures such as segmentation and mass slicing were used, which will prolong the surgical time and inevitably cause the injury to the vagina, vaginal stump, or surrounding organs [25, 26]. Morcellator was initially used during the minimally invasive myomectomy. For experienced surgeons, it has the advantages of reducing the time of sample removal. Application of the morcellator to the sample removal of the large uterus can avoid or reduce the injury to the vagina, vaginal stump, and the time of sample removal and thus helps the patients recover [27, 28]. In this study, we observed significantly shorter mean operation time and uterus removal time and smaller incidence of intraoperative complications in the morcellation group than in the transvaginal group. These findings confirmed the above advantages of TLH through uterine morcellation.

Attention should be paid to procedures during TLH through transvaginal approach for large uterus as follows: (1) for patients with vaginal atrophy or poor vaginal elasticity, care should be taken to avoid the injury to the vagina and vaginal stump due to the exposure difficulties; (2) care should be also taken to avoid the injury to the bladder due to the repeated vaginal tractor, excessive traction, or poor exposure during the uterus morcellation. In this study, we observed 4 cases of vaginal stump laceration, 1 case of bladder injury, and 1 case of ureteral injury in transvaginal group. Our rates of bladder (0.62%) and ureteric (0.62%) injury are comparable to other studies reporting 1.0–1.8% bladder and 0.2–0.4% ureteric injuries [2]. The bladder injuries occurred due to poor exposure. They occurred while the surgeon was conducting uterine traction because the urinary bladder was mistaken as the uterine. The ureteral injury (ureterovaginal fistula) developed 7 d after surgery and underwent ureteral bladder transplantation. The patient was uneventful during the 1 year of follow-up. The vaginal stump laceration results from the injury to the vagina or vaginal stump due to the dissection or traction. The middle and inferior segment of the laceration (not dehiscence) of the vaginal stump in this study were sutured from the vagina whereas its upper segment can be sutured synchronously during the laparoscopic vaginal stump suture. This indirectly reflects the difficulties of transvaginal removal of uterine samples. To avoid the risk of laceration, the large uterus should be cut into patches repeatedly and violent downward retraction of large masses of samples should not be allowed. In addition, the surgeons should not lose patience due to the prolonging of the surgical time.

With regard to the vaginal stump suturing, in the past, TLH mainly applied the method of transvaginal stump suture after transvaginal uterus removal due to the immaturity and unpopularization of the laparoscopic suture technique. However, with the maturity of the laparoscopic suture technique, there was almost no difference regarding the cost of time for suturing the vaginal stump between transvaginal and laparoscopic suture techniques. Transvaginal suture is inferior to the laparoscopic suture in terms of organizational involution due to the exposure difficulties. Whereas the rough surface intertangles in the pelvic cavity during laparoscopic suture can avoid the risk of vaginal stump polyps due to the existence of the peritoneum, however, it may occur during the transvaginal suture because the rough surface is exposed in the vagina. Therefore, we suggest that the vaginal stump suturing in both surgeries should be performed under laparoscopy. In this study, the vaginal stump suture was all conducted under the direction of laparoscopy.

Uterine leiomyomata are the most common pelvic tumors experienced in women [29]. Minimally invasive hysterectomy has proven its benefits of cosmesis, lower blood loss, less pain, shorter length of hospital stay, and faster recovery [29]. Given the acknowledged advantages of minimally invasive approaches, surgeons continue to explore and develop surgical techniques in order to avoid laparotomy. Currently, uterine specimen removal through minimally invasive techniques has been feasible in those patients with enlarged uteri, which were previously performed via laparotomy. The occurrence of uterine morcellation has increased for this reason. Morcellation allows for removal of tissue fragments through a smaller abdominal wall incision or colpotomy. However, application of laparoscopic morcellation brings not only benefits but also potential risks, that is, mainly mechanical risks [30, 31]. Increasing evidence suggested that intra-abdominal specimen morcellation was associated with an increased risk of occult cancerous tissues spreading beyond the abdominal cavity, thus impacting negatively the prognosis of patients [32–34]. In April and November 2014, the US Federal Drug Administration (FDA) advises against the use of power morcellators, which increases concerns on the embrace of minimally invasive approach for myomectomy and hysterectomy (especially in case of large uteri or supracervical hysterectomy). On the contrary, another review by Stine et al. [35] suggests that morcellation is an effective method of specimen removal that can decrease the need for laparotomy. In addition, the Society of Gynecologic Oncology (https://www.sgo.org/newsroom/position-statements-2/morcellation/) suggested that it is generally contraindicated to morcellate a specimen in the presence of a documented malignancy or in a patient in whom malignancy is strongly suspected secondary to the potential dissemination into the abdominal cavity and however recommended the use of morcellation if malignant tumors can be excluded. In this study, preoperative and postoperative examinations showed that all lesions were all benign tumors without any evidence of malignant lesions. Our findings suggest the safety of morcellators in TLH and advantages in nonmalignant cases.

Similarly, uterus specimen extraction through the transvaginal approach also needs segmental resection, split-half resection, or piecemeal resection of the uterus according to uterus size and shape due to the limited operation field. Thus, partial cut surface of the uterine body will inevitably be exposed to the pelvic cavity. Meanwhile, fragmental tissue due to the piecemeal resection of the large uterine body will also inevitably be involved in the pelvic cavity. Therefore, if there existed uterine sarcomas, involvement of the abdominal and pelvic cavity may be unavoidable after transvaginal removal of large uterus. The key point of the question was to make a comprehensive preoperative evaluation which is very important to minimize or exclude any potential malignant tumor. In our series, all patients underwent preoperative workup including evaluation of detailed clinical history, blood test, electrocardiogram, pelvic and kidney ultrasonography, thoracic and abdominal X-ray examination, and CT or MRI examination to exclude the malignant lesions. Besides, patients were adequately counseled to make an informed decision regarding undergoing a morcellation procedure. In this study, preoperative examinations showed that all lesions were benign tumors and postoperative histology examination showed 5 cases of cellular leiomyoma in morcellation. Furthermore, we performed an at least 5-year follow-up and observed no recurrence or malignant transformation in these cases. Our findings were consistent with the previous report. However, it also reminds us that, for patients with larger uteri who will undergo TLH through morcellation or transvaginal approaches, laparotomy should be considered if preoperative examinations show that there existed rapid growth of the myoma or abnormalities on the ultrasound or MR images. In addition, thorough washing of abdominal and pelvic cavity after surgery is vitally important for large uterine body no matter which extraction approach is adopted which can reduce maximally intra-abdominal spread of the undiagnosed uterine malignancies. However, it should also be noted that preoperative ultrasound or MR examinations are very important to minimize any potential tumor spread.

Two cases of conversion to laparotomy, one in each group, occurred. They occurred while the difficulty of surgical exposure was encountered by surgeons due to huge uterine size. We did not consider these laparoconversions as a proper complication as we believe, in agreement with other authors [22], that the cause of conversion, not the conversion itself, may be the major complication. We had laparoconversions in 2 procedures, not for intraoperative complications but for difficult vision and anesthesiologic problems, so we consider our approach as prudent, aimed at avoiding an additional risk for the patients.

## 5. Conclusions

TLH through uterine morcellation can reduce the operation time, uterus removal time, and the intraoperative complications and provide comparable postoperative outcomes compared to that through the transvaginal approaches. For experienced surgeons, the use of uterine morcellator is worth advocating.

## Figures and Tables

**Table 1:** Baseline data in two groups.

	Transvaginal group (*n* = 162 cases)	Morcellation group (*n* = 254 cases)	*P* values
Age (years)	52.4 ± 4.2	51.6 ± 4.8	0.08
BMI (kg/m^2^)	24.6 ± 2.2	24.3 ± 2.4	0.20
Uterine size (g.w.)	14.6 ± 2.3	14.3 ± 2.6	0.23
Parity (times)	1.4 ± 1.2	1.5 ± 1.3	0.43
Parous (%)			0.973
Yes	158 (97.5)	248 (97.6)	
No	4 (2.5)	6 (2.4)	
Previous cesarean section	39	53	0.44
Uterus dimensions with ultrasound, mm			0.23
Longitudinal axis	15.2 ± 1.2	15.7 ± 1.4	
Transverse axis	11.6 ± 1.3	11.2 ± 1.1	
Indications for TLH			
Fibroid	107	151	0.18
Adenomyoma associated with menorrhagia and dysmenorrhea	55	103	0.18

TLH: total laparoscopic hysterectomy and BMI: body mass index.

**Table 2:** Intraoperative outcomes in two groups.

	Transvaginal group (161 cases)	Morcellation group (*n* = 253 cases)	*P* values
Operation time (min)	128.6 ± 56.4	116.4 ± 44.6	0.02
Uterus removal time (min)	20.8 ± 7.8	15.8 ± 6.6	0.00
Blood loss (mL)	122.8 ± 61.4	113.2 ± 56.4	0.10
Uteri weight (g)	601.8 ± 138.4	612.4 ± 143.8	0.46
Length of hospital stay (days)	3.8 ± 1.4	3.6 ± 1.6	0.19
Conversion to laparotomy	1 (0.62%)	1 (0.39%)	0.933
Intraoperative complications	12 (7.5%)	6 (2.4%)	0.023

**Table 3:** Postoperative complications in two groups.

	Transvaginal group (161 cases)	Morcellation group (*n* = 253 cases)	*P* values
Pelvic hematoma	2 (1.2%)	1 (0.4%)	0.69
Vaginal stump infection	3 (1.9%)	6 (2.4%)	1.00
Lower limb venous thrombosis	1 (0.6%)	0 (0)	0.82
